# Evidence for a Functional Link Between the Nrf2 Signalling Pathway and Cytoprotective Effect of S-Petasin in Human Retinal Pigment Epithelium Cells Exposed to Oxidative Stress

**DOI:** 10.3390/antiox14020180

**Published:** 2025-02-04

**Authors:** Michela Pizzoferrato, Giacomo Lazzarino, Anna Brancato, Elisabetta Tabolacci, Maria Elisabetta Clementi, Giuseppe Tringali

**Affiliations:** 1 Pharmacology Section, Department of Health Care Surveillance and Bioethics, Università Cattolica del Sacro Cuore, Largo F. Vito 1, 00168 Rome, Italy; michela.pizzoferrato@unicatt.it; 2 Fondazione Policlinico Universitario Agostino Gemelli IRCSS, 00168 Rome, Italy; elisabetta.tabolacci@unicatt.it; 3 Departmental Faculty of Medicine, UniCamillus-Saint Camillus International University of Health Sciences, Via Di Sant’Alessandro 8, 00131 Rome, Italy; giacomo.lazzarino@unicamillus.org; 4 Department of Health Promotion, Mother and Child Care, Internal Medicine and Medical Specialties of Excellence “G. D’Alessandro”, University of Palermo, 90127 Palermo, Italy; anna.brancato@unipa.it; 5 Dipartimento di Sanità Pubblica e Scienze della Vita, Sezione di Medicina Genomica, Università Cattolica del Sacro Cuore, Largo F. Vito 1, 00168 Rome, Italy; 6 Istituto di Scienze e Tecnologie Chimiche “Giulio Natta” SCITEC-CNR, Largo Francesco Vito 1, 00168 Rome, Italy; elisabetta.clementi@scitec.cnr.it

**Keywords:** S-Petasin, ARPE-19 (human RPE cell line), nuclear factor erythroid 2-related factor (Nrf2), oxidative stress, apoptosis

## Abstract

The retinal pigment epithelium (RPE) is a highly specialised monolayer epithelium subjected to constant oxidative stress, which, in the long term, favours the development of a complex pathological process that is the underlying cause of macular damage. Therefore, counteracting the overproduction of ROS is the best-researched approach to preserve the functional integrity of the RPE. S-Petasin, a secondary metabolite extracted from the plant *Petasites hybridus*, has numerous biological effects, which highlight its anti-inflammatory and antioxidative properties. The aim of our study is to investigate whether S-Petasin exerts cytoprotective effects by protecting the RPE from oxidative damage. The effects of pretreatment with S-Petasin were assessed by the determination of the cell viability, intracellular ROS levels, activation of the Nrf2 pathway and the resulting post-transcriptional antioxidant/antiapoptotic response. Our results show that S-Petasin pretreatment (1) reduces intracellular ROS levels, improving cell viability of RPE exposed to oxidative damage; (2) activates the Nrf2 signalling pathway, modulating the post-transcriptional response of its antioxidant chemical biomarkers; (3) reduces the Bax levels, and an increase in those of Bcl-2, with a concomitant downregulation of the Bax/Bc-2 ratio. Overall, our results provide the first evidence that S-Petasin is able to protect the RPE from oxidative damage.

## 1. Introduction

Macular degeneration or maculopathy is a chronic and progressive pathology, characterised by the degeneration of the retinal tissue of the macula. The most common form of maculopathy is certainly the senile one linked to ageing (age-related macular degeneration; AMD) [1]. Based on the clinical–developmental characteristics, AMD can be classified into a non-neovascular form (dry or atrophic) and a neovascular form (wet or exudative). Current AMD pharmacological strategies only slow the progression of the disease. Recent research has identified and developed new approaches to reduce oxidative stress, chronic inflammation, the activity of toxic molecules (e.g., lipofuscin) and apoptosis, all factors that characterise the pathology of the disease, in an attempt to provide therapeutic alternatives. However, the use of drugs presents several pharmacokinetic problems, such as bioavailability, permanence in situ, frequency of administration and poor patient compliance [2,3]. Consequently, research is now focusing on gene and cell therapy, which appears to be a promising approach. However, immunogenicity and vector-mediated cytotoxicity can be a problem with these therapies [4]. Therefore, identifying new primary and secondary prevention strategies is of fundamental importance for AMD, since it is still a difficult to treat ocular pathology [5,6]. It is precisely from this perspective that measures, such as reducing exogenous risks (smoking, light and radiation) [7,8] or making use of non-pharmacological interventions (nutrition or supplements use), can have a significant impact on the prognosis and treatment of patients, even in advanced age [9]. In particular, clinical studies have shown that the continued use of high-dose multivitamins and antioxidants can decrease the risk of AMD progression by 25% in 5 years, especially in subjects with high-risk factors [10,11].

The retinal pigment epithelium (RPE), in its role as a blood–retinal barrier, plays an important role in the pathophysiology of the retina, providing metabolic and functional support to the photoreceptor outer segment [12]. Degenerative changes of the RPE at the cellular level caused by oxidative damage and the accumulation of undegraded material, such as lipofuscin and drusen, can impair its functionality [13,14,15]. In this context, an adequate supply of antioxidants, particularly of natural origin, can be an important support to slow and delay the “switch” of the RPE from physiological to pathological conditions, generated by the imbalance between ROS and antioxidant defences [16].

*Petasites officinalis* (syn. *Petasites hybridus*) is a medicinal plant that has been used in folk medicine for centuries, particularly for its antispasmodic and anti-asthmatic properties [17,18]. Recent studies have shown that extracts from different *Petasites* species have therapeutic effects on migraine- and headache-associated pain, as well as neuroprotective and anticancer effects, in vivo and in vitro [19,20,21,22]. Among the metabolites extracted from the plant, which are assumed to have therapeutic activity, the attention of researchers in recent years has focused, in particular, on four sesquiterpenoids: Petasin, S-Petasin and their isomers, into which they, respectively, convert spontaneously: IsoPetasin and S-IsoPetasin [23]. S-Petasin, the sesquiterpene methylthio derivative of Petasin isolated from the leaves and roots of different species of *Petasites*, is the compound that has most attracted researchers’ attention in recent years, thanks to its numerous pharmacological properties in vivo and in vitro that involve different molecular mechanisms [18,24,25,26,27]. Interestingly, while the mechanisms of action underlying the cytoprotective, anti-inflammatory and anti-migraine effects of S-Petasin (both as a phytocomplex and as a single active compound extracted from the plant) have been well characterised, little is still known about its antioxidant activity. In fact, although many studies have described the antioxidant potential of different plant extracts, rich in polyphenols and sesquiterpenes [28,29], few of those studies have investigated in detail the antioxidant effect of the single active ingredients present in *Petasites* extracts [30,31,32].

This said, the present study has been designed with the aim of investigating whether S-Petasin has antioxidant properties and, at the same time, if it is able to protect the RPE from oxidative damage, which underlies many degenerative eye diseases. For this purpose, we have utilised an oxidative stress experimental model, already developed and validated in precedent studies, involving the use of a RPE human cell line exposed to H_2_O_2_ [33,34,35]. The results of the study led to the hypothesis that S-Petasin may strengthen the endogenous antioxidant defences through activation of antioxidant response elements linked to the Nrf2 signalling transduction pathway.

## 2. Materials and Methods

### 2.1. Reagents

S-Petasin *Petasites* japonicus (sweet coltsfoot) was purchased from Sigma-Aldrich and dissolved in DMSO (Sigma-Aldrich, St. Louis, MO, USA) to obtain a 10 mM stock solution; further dilutions to the working concentrations were made in an incubation medium. The diluted DMSO (1/1000) did not interfere with the experimental procedures or cell viability. Ascorbic acid (vit C) and hydrogen peroxide (H_2_O_2_) were purchased from Sigma-Aldrich. Ascorbic acid was first solubilised in water (stock solution) and then added to the culture medium, while H_2_O_2_ was diluted directly in the culture medium.

### 2.2. Cell Cultures

ARPE-19 cells were purchased from the American Type Cell Culture (ATCC-CRL-2302, Manassas, VA, USA) and cultured according to the manufacturer’s instructions. The cells were cultured in a DMEM/F12 medium (Sigma-Aldrich, St. Louis, MO, USA) supplemented with 10% FBS (Gibco; Thermo Fisher Scientific Inc., Waltham, MA, USA), 2 mM L-Glutamine (Sigma-Aldrich, St. Louis, MO, USA) and 100 U/mL penicillin–streptomycin (Thermo Fisher Scientific Inc., Waltham, MA, USA) at 37 °C in a 5% CO_2_ environment. When 80% confluency was reached, cells were split and subcultured at a concentration of 30,000 cells/ cm^2^, while, for all the experiments, the cells were plated at a density of 15,000 cells/well.

### 2.3. Cell Viability

The cells, in starvation conditions (0% FBS), were treated for 24 h with plain medium (control group) or S-Petasin, range 0.01–10 µM, or, alternatively, with 750 µM of ascorbic acid, chosen as a positive control, to assess the antioxidant response in our experimental paradigm. After this pretreatment period, the cells were exposed to oxidative stress with 300 µM H_2_O_2_ for a further 24 h. At the end of the experiment, cell viability was assessed by the MTS assay (Promega, Waltham, MA, USA), performed according to the manufacturer’s instructions. A microplate photometer (Victor 4, PerkinElmer, Waltham, MA, USA) was used to measure the fluorescence at 485/535 nm of excitation/emission. The results were presented as the mean ± SEM of three independent experiments performed in quadruplicate (12 replicates per experimental group) and expressed as a percentage of the untreated control.

### 2.4. ROS Detection

The cells were plated in 96-well multi-wells at a density of 15,000 cells/well. Twenty-four hours after plating, the cells were pretreated with S-Petasin in a concentration range of 0.01–10 µM under starvation conditions. Following the pretreatment, the cells were exposed to damage with H_2_O_2_ for another 24 h. At the end of the experiment, ROS production was determined immediately. Measurement of the ROS intracellular levels was performed using the Abcam ROS kit for the quantification of ROS (DCFDA—Cellular ROS Assay Kit; Abcam ab113851), according to the manufacturer’s instructions. DCFDA taken up by the cells is oxidised by reactive oxygen species with a fluorescent readout (Ex/Em 485/535 nm) by a microplate photometer (Victor 4, PerkinElmer, Waltham, MA, USA). The results were expressed as a fold increase compared to the untreated control.

### 2.5. Quantification of Intracellular Nrf2 Levels

Cells were plated in 96-well plates at a seeding density of 15,000 cells/well, following the original experimental design described above. At the end of the experiments, the intracellular levels of Nrf2 were determined using a cell-based colorimetric ELISA kit (LSBio, LifeSpan Biosciences; Seattle, WA, USA), according to the manufacturer’s instructions [36]. Briefly, cells are incubated with primary antibodies (rabbit anti-Nrf2 or mouse anti-GAPDH; the latter used as an internal positive control), followed by an overnight incubation at 4 °C. After this overnight incubation, secondary antibodies (included in the kit; anti-rabbit IgG for Nrf2 and anti-mouse IgG for GADPH) conjugated to peroxidase were added, and then, the samples were read with a microplate reader (BioTek™ Elx800 -Box 998; BioTek Instruments, Winooski, VT, USA) [optical density (OD) at 450 nm]. Positive controls were performed in the same plates as the target experiments. All values obtained, normalised to the OD450 of GAPDH, were expressed as a percentage relative to the control (untreated cells). Interestingly, the use of ELISA assays is now considered a valid alternative tool for the quantitative measurement of Nrf2 levels in whole cells [37].

### 2.6. Bax and Bcl-2 Detection

To determine the possible antiapoptotic effects associated with S-Petasin pretreatment, we measured, in the presence of oxidative damage, the protein levels of two pro- and antiapoptotic factors, respectively, Bax and Bcl-2. A colorimetric cell ELISA kit from Assay Biotechnology (Sunnyvale, CA, USA) was used to measure the intracellular levels of the Bax and Bcl-2 proteins, according to the manufacturer’s instructions [38]. The samples were read at 450 nm using a microplate photometer (Victor 4, PerkinElmer, Waltham, MA, USA). All absorbance values obtained were corrected for housekeeping, and the results were expressed as a percentage compared to the untreated control.

### 2.7. Sample Processing for Metabolic Analysis

RPE cells from different experimental conditions were washed twice with ice-cold PBS and centrifuged at low speed (1860× *g*) for 5 min at 4 °C. After the last washing, cell pellets were deproteinised by adding 1 mL of an ice-cold deproteinising solution composed of CH_3_CN + 10 mM KH_2_PO_4_ (3:1; *v*:*v*), pH 7.40, according to a deproteinisation procedure previously set up [39]. After vortexing for 60 s, the samples underwent a high-speed centrifugation at 20,690× *g* for 10 min at 4 °C. The supernatants were washed with large volumes of chloroform (three times the sample volume) to remove the organic solvent, centrifuged again, as indicated above, and the upper aqueous phases were withdrawn and saved at −80 °C until analysed by HPLC. The processing method is suitable to perform the chromatographic separation of several low molecular weight metabolites, including representative biomarkers of oxidative/nitrosative stress (malondialdehyde, NO_2_ and NO_3_); reduced glutathione (GSH), as the main intracellular antioxidant (reduced glutathione, GSH) and compounds related to its metabolism (NADP^+^ and NADPH).

### 2.8. HPLC Analysis of Intracellular Antioxidants and Biomarkers of Oxidative/Nitrosative Stress

The HPLC apparatus consisted of a Surveyor HPLC system (Thermo Fisher Italia, Rodano, Milan, Italy) coupled to a highly sensitive diode array, equipped with a 5 cm light path flow cell and set up between 200 and 350 nm in wavelength. Data acquisition and analysis were performed by a PC using the ChromQuest^®^ 5.0 software package provided by the HPLC manufacturer. Separation and quantification of the intracellular antioxidants and biomarkers of oxidative and nitrosative stress were carried out on 100 µL of deproteinised cell extracts using a Hypersil C-18, 250 × 4.6 mm, 5 µm particle size column (Thermo Fisher Scientific) by applying an ion-pairing chromatographic method set up in our laboratory and described in detail elsewhere [40]. Identification and quantification of the intracellular compounds were carried out at a 206 nm wavelength, excluding malondialdehyde and NADP^+^ (260 nm wavelength), as well as NADPH (340 nm wavelength), by comparing peak areas and the absorption spectra of unknown samples with those of runs of ultrapure standard mixtures with known concentrations. In each cell extract, the total amount of proteins was determined according to the Bradford method [41]. All the concentration values of the metabolites of interest were normalised for the total cell protein content and expressed as nmol/mg of proteins.

### 2.9. Statistical Analysis

Each experiment was repeated two or three times, according to a randomised block design. The data obtained were analysed by means of a two-way analysis of variance (ANOVA) for the factors “time” and “treatment”; when no significant differences were found among the different experiments, performed with the same experimental protocol, a full analysis of all samples was considered justified. All experiments were performed with no less than six replicates per experimental group. All results were expressed as mean ± 1 standard error of the mean (SEM) of (n) replicates per experimental group. Data were subsequently analysed by one-way ANOVA with Newman–Keuls post hoc for comparisons of group means or by Dunnett’s test, when appropriate, using a computer programme: Prism^TM^ (GraphPad, San Diego, CA, USA). Differences were considered statistically significant if *p* < 0.05.

## 3. Results

### 3.1. S-Petasin Cytoprotective Effects

The first series of experiments was performed to determine the cytoprotective effect of S-Petasin in our experimental model of RPE cells, both in basal and stimulated conditions. Based on our experience, we treated the cells with H_2_O_2_ to mimic oxidative damage [34,35]. The used concentration of H_2_O_2_ (300 µM) was chosen based on dose–response experiments previously performed [30,31]. Furthermore, we tested and compared the S-Petasin effect with that of vitamin C (750 µM), considered as a positive control in our experimental paradigm. Indeed, the antioxidant properties of vitamin C in RPE cells, exposed to oxidative stress, are well demonstrated in the literature [42,43].

**Figure 1:**
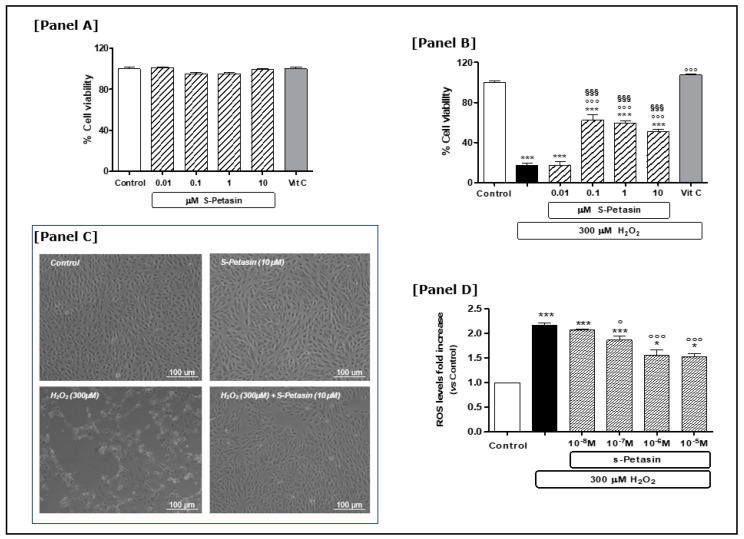
**S-Petasin protective effects on ARPE-19 cells.** S-Petasin does not show protective effects on cellular viability under baseline conditions (**A**); on the contrary, S-Petasin (concentration range 0.1–10 µM) improves cell viability if administered 24 h before oxidative damage induced by H_2_O_2_ (**B**). The observed results with S-Petasin are comparable to those of vitamin C (vit C), which has been used as an internal control in our experimental cell model (see text). (**C**) Representative phase contrast microscopy images of human ARPE-19 cells in the different experimental conditions. (**D**) The ability of S-Petasin pretreatment to reduce H_2_O_2_-induced ROS levels. The results (**A**,**B**) are expressed as a percentage relative to the control and are presented as the mean ± SEM of data from three separate experiments; each experiment was performed in quadruplicate. The ROS intracellular levels (**D**) are expressed as fold increases in respect to the control, and they are presented as the mean ± SEM of three replicates per experimental group from three independent experiments. One-way ANOVA followed by post hoc Newman–Keuls was carried out. * = *p* < 0.05 and *** = *p* < 0.001 vs. control; ° and °°°: *p* < 0.05 and *p* < 0.001 vs. H_2_O_2_ alone, respectively. ^§§§^ = *p* < 0.001 vs. vitamin C (vit C).

Having identified appropriate doses and a timescale for determining oxidative damage, we subsequently investigated the potential protective effect of S-Petasin under both basal and oxidative stress conditions. The concentrations of S-Petasin used in our experimental paradigm to determine the extent of the cytoprotective effect, and thus to derive the dose–response curve, cover a wide range of values from 0.01 to 10 µM. The choice of concentrations also took into account what has already been reported in the literature on different experimental cell models. Under basal conditions, treatment with S-Petasin did not alter ARPE-19 cell viability at any of the concentrations tested. Notably, no change in cell viability was observed when S-Petasin supplementation for 24 h (range 0.01–10 µM) was compared to the control group or to vitamin C [Figure 1A]. This ensured that the doses subsequently used in our experimental paradigm were not toxic to EPR cells. Contrary to what was observed under basal conditions, the addition of H_2_O_2_ for 24 h caused a significant reduction in cell viability of 83% [Figure 1B]. These reductions were counteracted by 24 h pretreatment with S-Petasin at the concentrations previously tested in the basal experiments (range 0.1–10 μM) [Figure 1B]. The observed protective effect of S-Petasin was statistically significant starting at 0.1 µM, whereas the lowest concentration tested (0.01 µM) did not alter the oxidative damage. No significant changes were observed among the different doses of S-Petasin that were effective in counteracting H_2_O_2_-induced oxidative damage [Figure 1B]. Vitamin C also reversed H_2_O_2_-induced damage in the same experimental paradigm, confirming the validity of both the experimental model and the observed effects of S-Petasin. Interestingly, the improvement in viability by pretreatment with S-Petasin, while highly significant, did not completely restore cell viability and was lower than with vitamin C [Figure 1B]. The findings obtained were also supported by the morphological observations performed after each experiment [Figure 1C].

An imbalance of the normal cellular redox state causes toxicity characterised by high ROS levels, oxidative damage and consequent cellular death. In this regard, previous studies have demonstrated that ARPE-19 cells exposed to high H_2_O_2_ concentrations undergo a reduction in viability as a result of an increase in ROS at both the cytoplasmic and mitochondrial level [35,36,44,45,46,47]. Therefore, subsequent experiments were conducted with the aim of observing whether S-Petasin could antagonise the increase in ROS intracellular levels induced by a high H_2_O_2_ concentration. As expected, exposure to H_2_O_2_ increased the ROS levels about twofold, which were antagonised by pretreatment (24 h) of S-Petasin in a dose-dependent manner [Figure 1D].

### 3.2. S-Petasin Increases Intracellular Nrf2 Levels and Modulates Post-Transcriptional Processes

**Figure 2:**
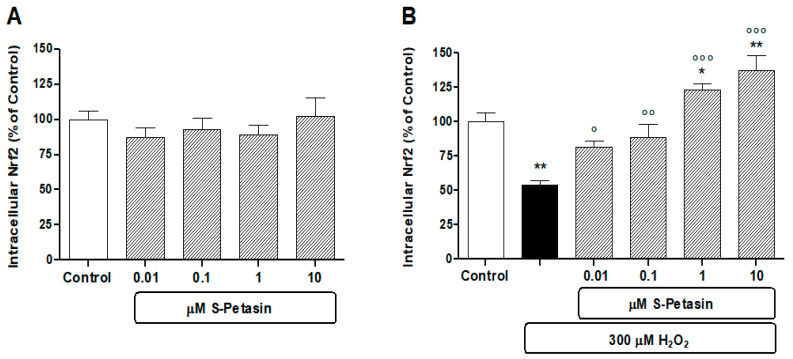
**S-Petasin modulates the** Nrf2 **levels.** The effects of S-Petasin on the intracellular Nrf2 levels under basal (**A**) and H_2_O_2_-stimulated (**B**) conditions. The results are presented as a percentage of protein compared to the untreated control (arbitrarily set at 100%). Data, from three independent experiments, are expressed as the mean ± SEM of 12 replicates per experimental group. * = *p* < 0.05 and ** = *p* < 0.01 vs. control; ° = *p* < 0.05, °° = *p* < 0.01 and °°° = *p* < 0.001 vs. H_2_O_2_ alone.

Nrf2 is a transcriptional factor that modulates the basal and stress-inducible responses. Today, the Nrf2/KEAP1 signalling pathway is considered the primary “sensor” of oxidative stress regulating redox homeostasis. Consequently, at this point in our study, it seemed interesting to study whether there was a correlation between the cytoprotective action of S-Petasin and the increase in cytoplasmic Nrf2 levels. With this in mind, we measured the intracellular levels of the Nrf2 protein after pretreating (24 h) with S-Petasin the cells in the presence or absence of H_2_O_2_-induced oxidative damage. Figure 2 shows that H_2_O_2_ reduces Nrf2 levels in our experimental paradigm; this effect is antagonised by 24 h pretreatment with S-Petasin [Figure 2B]. On the contrary, in the absence of oxidative damage, the pretreatment with S-Petasin showed no effect at the doses used [Figure 2B]. It therefore appears that the protective effect of S-Petasin only occurs in the presence of oxidative stress, facilitating the increase in intracellular levels of Nrf2 and subsequent nuclear translocation.

**Figure 3:**
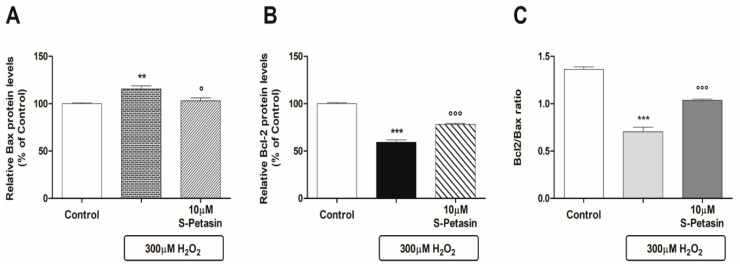
**S-Petasin regulates the Bax and Bcl-2 levels in ARPE-19 cells exposed to oxidative damage.** S-Petasin downregulated the protein levels of Bax (**A**) and upregulated those of Bcl-2 (**B**), as well as increasing the Bcl-2/Bax ratio (**C**). (**A**,**B**) The means ± SEM of two independent experiments performed in quadruplicate (eight replicates per experimental group). (**C**) The cytoprotective effect of S-Petasin like the Bcl-2/Bax ratio: values are expressed as the ratio of the means of Bcl-2 and Bax in absolute values. ** *p* < 0.01 and *** *p* < 0.001 vs. control; ° *p* < 0.01 and °°° *p* < 0.001 vs. H_2_O_2_ alone.

Subsequent studies were conducted with the aim of investigating both the antioxidant and apoptotic post-transcriptional mechanisms that could be implicated in the stimulation of Nrf2 by S-Petasin. Today, we know that Nrf2-mediated Bcl-2 protein regulation plays a central role in the antiapoptotic response of cells to exo/endogenous oxidative stress. Therefore, we measured the intracellular Bax and Bcl-2 levels in the presence of H_2_O_2_ with and without pretreatment with S-Petasin. H_2_O_2_ caused, on the one hand, an increase in the intracellular levels of the Bax protein and, on the other hand, a reduction in the Bcl-2 protein levels. The observed effects were significantly reversed where the cells were pretreated with S-Petasin for 24 h [Figure 3A,B]. In addition, Figure 3C shows the Bcl-2/Bax ratio, which optimally represents the antiapoptotic index and, thus, the cytoprotective efficacy of S-Petasin. Cells exposed to H_2_O_2_ showed a reduction in the ratio of approximately 48% compared to the untreated cells, which decreased to 24% after pretreatment with S-Petasin.

Nrf2 also regulates several cytoprotective genes that participate in the cellular antioxidant response, which expression are induced in response to cellular stress [48]. Therefore, we investigated the Nrf2 biochemical effects, which are downstream of the antioxidant post-transcriptional response. Using the experimental paradigm previously described, we examined in the presence or absence of S-Petasin, both under basal conditions and in the presence of oxidative damage, the levels of glutathione (GSH), a non-enzymatic antioxidant; of malondialdehyde (MDA), an indicator of lipid peroxidation; and of nitrates (NO_3_^−^) and nitrites (NO_2_^−^), as well as of the NADP^+^/NADPH ratio. Table 1 summarises the results obtained: (a) S-Petasin significantly increased the GSH levels under oxidative stimulus but not in basal conditions; (b) S-Petasin decreased in a statistically significant manner the MDA concentrations in the presence of H_2_O_2_; (c) the intracellular nitrate levels were significantly reduced after pretreatment with S-Petasin in both experimental conditions; in contrast, the nitrite levels were reduced only in the presence of oxidative damage; (d) after pretreatment with S-Petasin, the NADP^+^/NADPH ratio was significantly reduced under oxidative damage conditions, whereas it increased under basal conditions.

The findings described above support our initial hypothesis that S-Petasin has antioxidant properties.

## 4. Discussion

The present study confirms and reinforces the hypothesis that S-Petasin, a secondary metabolite extracted from the leaves, flowers and roots of various *Petasites* species, is a nutraceutical substance with high antioxidant potential, which has an effective cytoprotective action in the presence of oxidative damage, the main cause of RPE ageing. Though there is various evidence about the possible therapeutic properties of S-Petasin and the mechanisms underlying these effects, this study shows, for the first time, the cytoprotective/antioxidant action of this substance at the level of the RPE.

Over the years, the awareness that the RPE plays a role of fundamental importance for the homeostasis of the retina, regulating the metabolic exchanges between the photoreceptors and the underlying choroid, has increasingly grown [49]. During ageing, RPE cells are no longer able to metabolise, and the products of degradation begin to accumulate (drusen), causing the thickening of Bruch’s membrane and nutritional trophic insufficiency, with consequent suffering of the photoreceptors [14,50]. This degenerative process is the beginning of the pathological picture characteristic of maculopathy. RPE cells are particularly vulnerable to the toxicity of high concentrations of ROS caused by chronic intracellular oxidative stress, although they show a greater adaptive response and resistance to oxidative damage compared to other cellular types [51]. Under physiological conditions, RPE cells have integrated antioxidant defence systems, enzymatic and non-enzymatic, which are usually effective in blocking the harmful effects of ROS. However, under pathological conditions sustained by endogenous and/or exogenous factors (e.g., age, metabolic diseases, smoking, environmental factors and exposure to UV light), an overproduction of ROS can be observed with a switch from physiological to pathological conditions [50,52]. Therefore, the attempt to counteract ROS overproduction is one of the most studied approaches to preserve the functional integrity of the RPE. In this context, studies on the use of natural products and herbal extracts as adjuvant antioxidant therapies to standard therapies are increasing.

Interestingly, while the current knowledge attributes several biological effects to the active metabolites of various *Petasites* species, including anti-inflammatory, anti-allergy, anti-asthmatic, anti-migraine, anti-tumour and neuroprotective activities, there is still conflicting evidence on the pro- or antioxidant effects of these substances. In fact, to date, experimental evidence shows both actions pro-oxidant/apoptotic on tumour cell lines [27,53] and antioxidant/cytoprotective on normal cell lines and on ex vivo/vitro experimental models [20]. The above is also valid for the single secondary metabolite S-Petasin [22,26]. However, many of these studies were conducted using phytocomplexes or metabolite extracts of various *Petasites* species rather than individual therapeutic agents. Therefore, we used a commercial S-Petasin in our study to avoid interferences related to the extraction procedures and/or other substances present in the extract. Moreover, we compared the S-Petasin effects with that of vitamin C (ascorbic acid), considered a positive antioxidant control in cell ARPE-19 experimental models [42,54]. To do this, we used the most effective concentration of vitamin C able to completely reverse H_2_O_2_-induced oxidative damage in our experimental paradigm. This allowed us, on the one hand, to validate the experimental system and, on the other hand, to determine how effective S-Petasin was compared to well-known antioxidants. By correlating the results on cell viability and the morphological ones obtained in our study, we can state that S-Petasin is a natural substance capable of protecting RPE cells from oxidative damage by reducing the ROS cytoplasmic levels induced by exogenous oxidative stress. In particular, the cytoprotective effect of S-Petasin was not observed under basal conditions [Figure 1A] but only in the presence of induced oxidative stress [Figure 1B]. Under these conditions, the presence of H_2_O_2_ significantly reduced RPE viability and increased the cytoplasmic ROS levels [Figure 1B,D]. This state of induced oxidative stress was counteracted by pretreatment with S-Petasin, which improved cell viability up to fourfold compared to the conditions characterised by the presence of H_2_O_2_ alone [Figure 1B]. The same trend was observed with vitamin C, which increased cell viability up to sixfold [Figure 1B].

A chronic increase in oxidative stress causes widespread cellular damage, characterised by an excessive release of ROS, which is normally counteracted by an increased inducible expression of the innate antioxidant and detoxification system. Cellular resistance to oxidative stress is correlated with a particular molecular phenotype: a high expression of antioxidant enzymes and DNA repair systems, as well as reduced apoptosis and activation of cell cycle arrest-inducing genes. The Nrf2 protein seems to play a decisive role in the appearance of the oxidative stress-resistant phenotype. Indeed, Nrf2 is an important transcription factor for the oxidative stress response and is considered the primary oxidative stress sensor that regulates redox homeostasis [53]. The Nrf2 signalling pathway relies on the nuclear availability of Nrf2 itself. In fact, under homeostatic conditions, the Nrf2 levels are normally low. This is because Nrf2 is bound to KEAP1, which promotes its ubiquitination and degradation. However, in the presence of intracellular ROS overproduction, the Nrf2 levels increase as Nrf2 escapes KEAP-1 binding and is free for nuclear translocation, where it regulates the transcriptional activation of its target genes [37,48,55,56]. In this respect, our results are very interesting, because they show how S-Petasin, in the presence of oxidative damage, stimulates the Nrf2 pathway, which manifests itself in a Nrf2 intracellular level increase and subsequent transcription of genes and events that ultimately protect RPE cells from oxidative damage. Effectively, the results of our study show that S-Petasin is able to modulate the levels of several biomarkers of oxidative stress: on the one hand, this reduces the intracellular levels of MDA, NO_2_^−^ and NO_3_^−^, as well as the NADP^+^/NADPH ratio, and, on the other hand, increases those of GSH, respectively decreased and increased by the oxidative damage induced by H_2_O_2_ in our experimental model. These results are also supported by the scientific literature, which highlights the ability of terpene and terpenoid compounds to act both as direct antioxidants through free radical scavenging mechanisms and as indirect antioxidants by enhancing the antioxidant state (enzymatic and non-enzymatic) [28,29,57,58].

Under physiological conditions, growth factors or survival signals stimulate the expression of antiapoptotic proteins belonging to the Bcl-2 protein family, which, by interacting with mitochondrial membranes, prevent the activity of proapoptotic factors like proteins of Bax and the Bak family. However, a variety of cellular stresses or exogenous damaging signals can activate the so-called “intrinsic pathway”, causing the permeabilisation of the outer mitochondrial membrane and the subsequent release of proapoptotic factors into the cytoplasm. In this regard, it is noteworthy to mention that the induction of Nrf2 nuclear overexpression upregulates the Bcl-2 antiapoptotic protein and downregulates the Bax proapoptotic protein [59]. With this in mind, we investigated if S-Petasin could also stimulate the “intrinsic pathway” by measuring the intracellular levels of Bax and Bcl-2. S-Petasin was able to improve the Bcl-2/Bax ratio in favour of antiapoptotic action, reinforcing our initial hypothesis regarding the ability of S-Petasin to protect RPE cells from H_2_O_2_-induced oxidative damage.

In conclusion, the above data provide a clear picture of the cytoprotective effects of S-Petasin on RPE cells under conditions of induced oxidative stress. However, our research undoubtedly has limitations and requires further investigation to strengthen the hypothesis of the cytoprotective effects of S-Petasin at the ocular level. Last, but not least, the cell line used in this study, hARPE-19, might present different karyotypes and pathophysiological characteristics than RPE in vivo [60,61]. Therefore, it will also be important to validate our observations in more physiological experimental models of RPE, both in vitro and in vivo. Nevertheless, hARPE-19 cells are still considered a valid experimental approach to study the molecular mechanisms of xenobiotics in the RPE phenotype, without heterotopic interference [62,63]. Within this context, it is understood that further studies will be necessary in the future to investigate the molecular mechanisms underlying the antioxidant effect of S-Petasin, which we cannot currently exclude. In particular, it will be of great interest to study the effects of S-Petasin as an inducer of Nrf2 and the “intrinsic pathway”, in order to understand whether the substance can also promote mitochondrial survival and have an antiapoptotic effect, in addition to its behaviour as a scavenger of ROS. Although some questions remain unanswered at the moment, the results of our “pilot study” lead us to hypothesise S-Petasin as a natural compound which supplementation, in addition to standard therapies, could reduce the RPE’s vulnerability to oxidative stress, the main cause of the ageing and the genesis of neurodegenerative retinal diseases. Therefore, our study acts as a “bridge” between basic and translational research, especially now that a part of the research in the field of neuro-ophthalmic pathologies is focusing on products and extracts of natural origin with antioxidant activity, due to their favourable safety profile and the possibility of long-term treatments.

## Figures and Tables

**Table 1:** The intracellular levels of GSH, MDA, nitrates (NO_3_^−^), nitrites (NO_2_^−^) and the NADP+/NADPH ratio in ARPE-19 cells both under basal (top) and oxidative stress conditions (bottom) in the presence of different concentrations of S-Petasin. Under basal conditions, the cells were treated with different concentrations of S-Petasin (range 0.1–10 µM) for 24 h. Instead, the experimental protocol of oxidative damage consisted of a pretreatment (24 h) with S-Petasin (range 0.1–10 µM) followed by 24 h of treatment with H_2_O_2_.

**Basal Conditions**					
***Biomarker***	***Control*** ***Mean ± SEM (n)***	***S-Petasin 10^−6^*** ***Mean ± SEM (n)***	***S-Petasin 10^−5^*** ***Mean ± SEM (n)***		
GSH	1.171 ± 0.101 (6)	1.022 ± 0.180 (6)	0.918 ± 0.029 (6)		
MDA	0.010 ± 0.001 (6)	0.009 ± 0.001 (6)	0.009 ± 0.002 (6)		
NO_2_^−^	1.190 ± 0.139 (6)	1.086 ± 0.074 (6)	1.092 ± 0.016(6)		
NO_3_^−^	30.29 ± 0.484 (6)	24.01 ± 2.413 (6) *	25.16 ± 0.207 (6) *		
NADP^+^/NADPH	0.667 ± 0.105 (6)	0.814 ± 0.077 (6)	0.970 ± 0.026 (6) *		
**Stress Conditions**					
***Biomarker***	***Control*** ***Mean ± SEM (n)***	***H_2_O_2_*** ***Mean ± SEM (n)***	***H_2_O_2_ +*** ***S-Petasin 10^−7^*** ***Mean ± SEM (n)***	***H_2_O_2_ +*** ***S-Petasin 10^−6^*** ***Mean ± SEM (n)***	***H_2_O_2_ +*** ***S-Petasin 10^−5^*** ***Mean ± SEM (n)***
GSH	1.171 ± 0.101 (6)	0.235 ± 0.038 (6) ***	0.813 ± 0.052 (6) ^°°°^	0.708 ± 0.068 (6) ^°°°^	0.705 ± 0.061 (6) ^°°°^
MDA	0.010 ± 0.001 (6)	0.027 ± 0.004 (6) ***	0.017 ± 0.003 (6) ^°°°^	0.019 ± 0.001 (6) ^°°^	0.020 ± 0.002 (6) ^°°^
NO_2_^−^	1.190 ± 0.139 (6)	2.014 ± 0.221 (6) ***	0.979 ± 0.112 (6) ^°°°^	0.856 ± 0.074 (6) ^°°°^	1.231 ± 0.096 (6) ^°°°^
NO_3_^−^	30.29 ± 0.484 (6)	43.22 ± 5.334 (6) ***	23.60 ± 1.880 (6) ^°°°^	30.40 ± 1.900 (6) ^°°^	32.77 ± 1.943 (6) ^°^
NADP^+^/NADPH	0.667 ± 0.105 (6)	5.551 ± 0.743 (6) ***	2.088 ± 0.240 (6) ^°°°^	1.533 ± 0.052 (6) ^°°°^	2.350 ± 0.197 (6) ^°°°^

The results are from two independent experiments. The data are expressed as nmol/mg protein, and the means ± 1 SEM of 6 replicates per group are shown. * = *p* < 0.05 and *** = *p* < 0.01 vs. control; ^°^ = *p* < 0.05, ^°°^ = *p* < 0.01 and ^°°°^ = *p* < 0.001 vs. H_2_O_2_ alone. GSH: glutathione; MDA: malondialdehyde.

## Data Availability

All data supporting the findings of this study are available within the article.
